# Therapeutic Effects of Decursin and *Angelica gigas* Nakai Root Extract in Gerbil Brain after Transient Ischemia via Protecting BBB Leakage and Astrocyte Endfeet Damage

**DOI:** 10.3390/molecules26082161

**Published:** 2021-04-09

**Authors:** Tae-Kyeong Lee, II-Jun Kang, Hyejin Sim, Jae-Chul Lee, Ji-Hyeon Ahn, Dae-Won Kim, Joon-Ha Park, Choong-Hyun Lee, Jong-Dai Kim, Moo-Ho Won, Soo-Young Choi

**Affiliations:** 1 Department of Biomedical Science and Research Institute for Bioscience and Biotechnology, Hallym University, Chuncheon 24252, Korea; tk-lee@hallym.ac.kr; 2 Department of Food Science and Nutrition, Hallym University, Chuncheon 24252, Korea; ijkang@hallym.ac.kr; 3 Department of Neurobiology, School of Medicine, Kangwon National University, Chuncheon 24341, Korea; janny20@naver.com (H.S.); anajclee@kangwon.ac.kr (J.-C.L.); jh-ahn@ysu.ac.kr (J.-H.A.); 4 Department of Physical Therapy, College of Health Science, Youngsan University, Yangsan 50510, Korea; 5 Department of Biochemistry and Molecular Biology, Research Institute of Oral Sciences, College of Dentistry, Gangnung-Wonju National University, Gangneung 25457, Korea; kimdw@gwnu.ac.kr; 6 Department of Anatomy, College of Korean Medicine, Dongguk University, Gyeongju 38066, Korea; jh-park@dongguk.ac.kr; 7 Department of Pharmacy, College of Pharmacy, Dankook University, Cheonan 31116, Korea; anaphy@dankook.ac.kr; 8 Division of Food Biotechnology, School of Biotechnology, Kangwon National University, Chuncheon 24341, Korea; jongdai@kangwon.ac.kr

**Keywords:** *Angelica gigas* Nakai, astrocyte endfeet, blood–brain barrier, decursin, hippocampus, immunoglobulin G, transient forebrain ischemia

## Abstract

*Angelica gigas* Nakai root contains decursin which exerts beneficial properties such as anti-amnesic and anti-inflammatory activities. Until now, however, the neuroprotective effects of decursin against transient ischemic injury in the forebrain have been insufficiently investigated. Here, we revealed that post-treatment with decursin and the root extract saved pyramidal neurons in the hippocampus following transient ischemia for 5 min in gerbil forebrain. Through high-performance liquid chromatography, we defined that decursin was contained in the extract as 7.3 ± 0.2%. Based on this, we post-treated with 350 mg/kg of extract, which is the corresponding dosage of 25 mg/kg of decursin that exerted neuroprotection in gerbil hippocampus against the ischemia. In addition, behavioral tests were conducted to evaluate ischemia-induced dysfunctions via tests of spatial memory (by the 8-arm radial maze test) and learning memory (by the passive avoidance test), and post-treatment with the extract and decursin attenuated ischemia-induced memory impairments. Furthermore, we carried out histochemistry, immunohistochemistry, and double immunohistofluorescence. Pyramidal neurons located in the subfield cornu ammonis 1 (CA1) among the hippocampal subfields were dead at 5 days after the ischemia; however, treatment with the extract and decursin saved the pyramidal neurons after ischemia. Immunoglobulin G (IgG, an indicator of extravasation), which is not found in the parenchyma in normal brain tissue, was apparently shown in CA1 parenchyma from 2 days after the ischemia, but IgG leakage was dramatically attenuated in the CA1 parenchyma treated with the extract and decursin. Furthermore, astrocyte endfeet, which are a component of the blood–brain barrier (BBB), were severely damaged at 5 days after the ischemia; however, post-treatment with the extract and decursin dramatically attenuated the damage of the endfeet. In brief, therapeutic treatment of the extract of *Angelica gigas* Nakai root and decursin after 5 min transient forebrain ischemia protected hippocampal neurons from the ischemia, showing that ischemia-induced BBB leakage and damage of astrocyte endfeet was significantly attenuated by the extract and decursin. Based on these findings, we suggest that *Angelica gigas* Nakai root containing decursin can be employed as a pharmaceutical composition to develop a therapeutic strategy for brain ischemic injury.

## 1. Introduction

Transient ischemia (TI) in the brain is induced by temporary hindrance of blood supply to the brain and evokes a transient ischemic attack, commonly known as a mini-stroke, that is a brief episode of neurological dysfunction without tissue death (infarction) [1]. TI inflicts ischemia-reperfusion injury onto the brain, and in particular, ischemia-reperfusion injury brings selective neuronal loss/death in vulnerable structures, such as the hippocampus, neocortex, and striatum [2,3,4,5]. It has been well accepted that ischemia-reperfusion for five minutes in gerbil forebrain brings neuronal loss/death selectively in the subfield cornu ammonis 1 (CA1) among hippocampal subregions (CA1–3) at 4–5 days after the ischemic insult; thus, this aspect of the neuronal loss/death is termed as “delayed neuronal death (DND)” [4,6]. Additionally, it is well accepted that the DND in the hippocampal CA1 is prone to be accompanied with impairments in spatial and learning memory [7,8].

It has been suggested that the mechanisms of DND following TI are complicated [9,10,11]. Among the mechanisms, oxidative stress triggered by excessive reactive oxygen species (ROS) production is considered as one of the mechanisms of DND [10,12]. In addition, excitotoxicity triggered by excessive accumulation of glutamate is considered as another [10,12]. Recently, besides, it has been reported that the disruption of the blood–brain barrier (BBB) following ischemic insults is one of the pathophysiological hallmarks of ischemic injury in the brain [13]. The central nervous system (CNS) is separated from blood vessels by the BBB, and the BBB enables the CNS to be maintained in a healthy condition with a highly selective semi-permeability [14].

**Figure 1:**
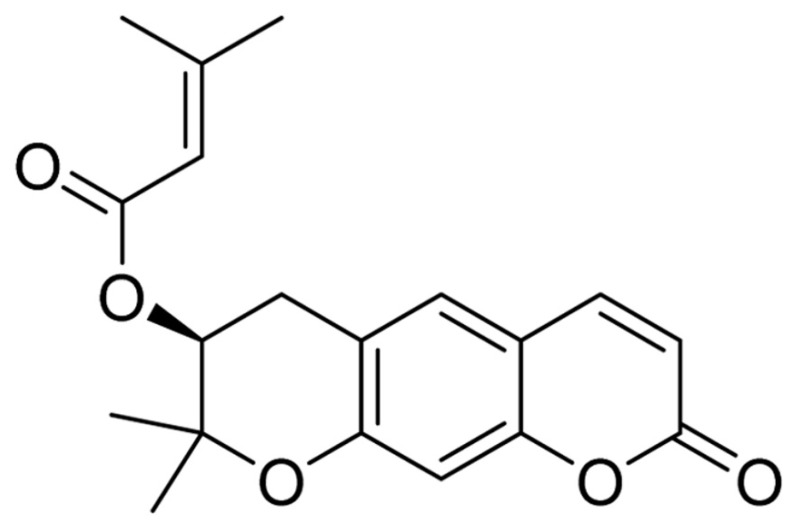
The chemical structure of decursin.

*Angelica gigas* Nakai (AGN) belongs to the Umbelliferae family, and its root has been used in oriental medicine to have beneficial properties such as anti-inflammatory and anti-amnesic activities [15,16]. The ingredients of AGN root extract (AGNE) have been well determined, as tabulated in Table 1 [17,18,19]. Among them, decursin, a coumarin derivative compound (Figure 1), is regarded as a major effective ingredient of AGNE and possesses advantageous attributes including anti-inflammatory activity, anti-glioblastoma activity, and neuroprotective effects [20,21,22]. Especially, for neuroprotection in ischemic brains, AGNE shows a neuroprotective effect via regulating angiogenesis in a rat model of transient focal cerebral ischemia, which is induced by occlusion of the middle cerebral artery [22]. In addition, decursin displays neuroprotective effects against amyloid β_25–35_-induced neurotoxicity in rat pheochromocytoma PC12 cells via suppressing mitochondrial apoptotic processes [23].

However, few studies on the neuroprotective effects of AGNE or decursin against TI injury in brains have been done. The major objective of this study, therefore, was to investigate protective effects in neuronal damage/death and spatial and learning memory impairment in gerbil hippocampus when AGNE and decursin was therapeutically administrated after TI in the forebrain. Furthermore, we examined TI-induced change in BBB integrity and leakage along with the alteration of astrocyte endfeet, which is a component of the BBB in the hippocampus after post-treatment with AGNE and decursin.

## 2. Results

### 2.1. Quantity of Decursin in AGNE

**Figure 2:**
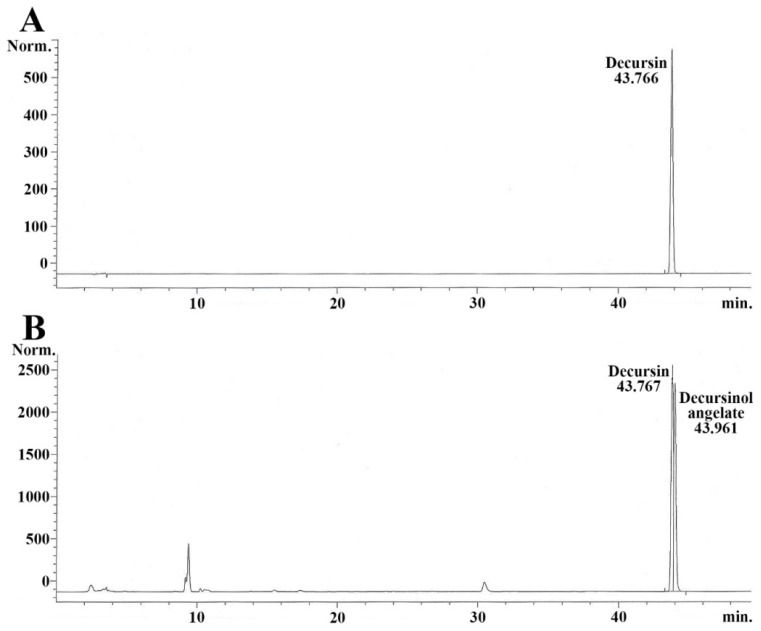
Representative HPLC chromatograms of standard decursin (**A**) and AGNE (**B**). Retention time of the standard decursin is 43.766 min and the retention times of the AGNE are 43.767 min (decursin) and 43.961 min (decursinol angelate), respectively.

The chromatogram for decursin (standard sample) was peaked at 43.766 min (Figure 2A). Decursin and decursinol angelate in the AGNE (test sample) were respectively detected at 43.767 and 43.961 min (Figure 2B). The decursin contained in the AGNE was quantified via calculating with each peak area. It contained approximately 7.3 ± 0.2%. The equation for the calibration curve of the standard sample (decursin) was “Area = 369,587.656x + 6.14 (x = mg/mL)”.

### 2.2. Dosage Determination of AGNE and Decursin for Neuroprotection against TI

In this experiment, in order to determine the dosage of AGNE showing neuroprotection, we orally treated vehicle (sterilized normal saline; 0.85% *w/v* NaCl), 200, 350, and 400 mg/kg of AGNE into the gerbils at 30 min after TI. Five days after TI, we examined the hippocampus by cresyl violet (CV) histochemistry, which is used to demonstrate the Nissl substance in cells.

**Figure 3:**
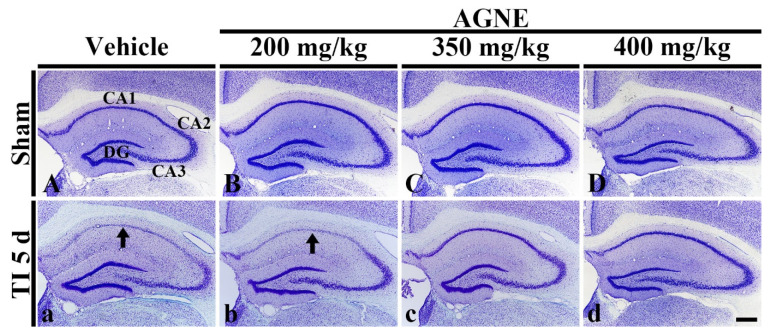
Cresyl violet (CV) histochemistry in the hippocampus of the sham/vehicle (**A**), sham/AGNE 200–400 mg/kg (**B**–**D**), transient ischemia (TI)/vehicle (**a**), TI/AGNE 200–400 mg/kg (**b**–**d**) groups at 5 days after TI. In the TI/vehicle and TI/200 mg/kg AGNE groups, CV stainability is weakened in CA1 (arrows) at 5 days after TI. On the other hand, in the TI/350 and 400 mg/kg AGNE groups, CV dyeability is similar to that in the sham/vehicle group. DG, dentate gyrus (*n* = 7 in each group). Scale bar = 400 μm.

All sham groups, cells in the hippocampus (CA1–3), were well stained by CV (Figure 3A–D). In the TI/vehicle group, cells of the stratum pyramidale (called pyramidal neurons or cells) in CA1, not CA2/3, showed weak CV dyeability at 5 days after TI (Figure 3a). In the TI/200 mg/kg AGNE group, CV dyeability in the pyramidal cells was similar to that in the TI/vehicle group at 5 days after TI (Figure 3b). On the other hand, in the TI/350 mg/kg and 400 mg/kg AGNE groups, CV dyeability in cells in all hippocampal subregions was similar to that in the sham/vehicle group at 5 days after TI (Figure 3c,d).

Based on this finding, we chose 350 mg/kg AGNE as an optimal dosage of neuroprotective effect (Figure 3). Additionally, considering the above HPLC analysis that the AGNE contained 7.3 ± 0.2% of decursin (Figure 3), the dosage of decursin was set as 25 mg/kg.

### 2.3. Attenuation of TI-Induced Cognitive Deficits by Decursin and AGNE

**Figure 4:**
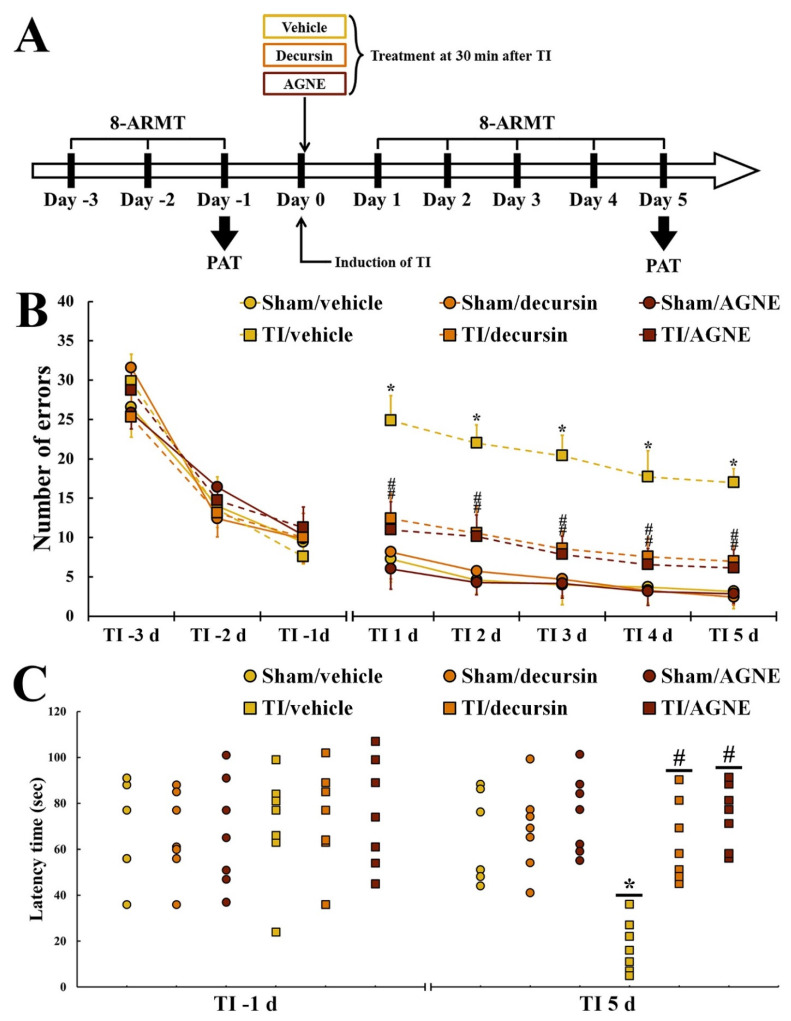
Experimental timeline (**A**), the mean number of errors in an 8-arm radial maze test (8-ARMT) (**B**) and latency time in a passive avoidance test (PAT) (**C**) in the sham/vehicle, sham/decursin, sham/AGNE, TI/vehicle, TI/decursin, and TI/AGEN groups at 3 days, 2 days, and 1 day before TI, and 1 day, 2 days, 3 days, 4 days, and 5 days after TI. The number of the errors in both TI/decursin and TI/AGNE groups are significantly reduced after TI compared with the TI/vehicle group. In addition, the latency time in the TI/decursin and TI/AGNE groups is significantly longer than that in the TI/vehicle group at 5 days after TI. The bars indicate the means ± standard error of mean (SEM) (*n* = 7 per group, * *p* < 0.05 versus sham/vehicle group, # *p* < 0.05 versus TI/vehicle group).

In order to investigate cognitive function, the gerbils in each group received an 8-arm radial maze test (8-ARMT) to examine spatial memory function at 3, 2, and 1 day before TI and 1, 2, 3, 4, and 5 days after, and a passive avoidance test (PAT) to investigate learning memory at 1 day before TI and 5 days after TI (Figure 4A).

#### 2.3.1. Spatial Memory

For three days before TI and sham operations, changes in the number of errors were not different among all groups, indicating that the gerbils were subjected to identical pre-training for 8-ARMT (Figure 4B).

In all sham groups, no significant difference in the numbers of errors was detected at 1 to 5 days after sham operations (Figure 4B). In contrast, the number of errors in the TI/vehicle group at one day after TI was significantly increased compared with that in the sham group (Figure 4B). At this time, however, in the TI/decursin and TI/AGNE groups, the number of errors was significantly decreased compared with that in the TI/vehicle group (Figure 4B). From three days after TI, the number of errors in the TI/decursin and TI/AGNE groups was more decreased, showing that the number of errors was significantly lower than that in the TI/vehicle group (Figure 4B).

#### 2.3.2. Learning Memory

At one day before TI, no significant difference in latency time was found among all groups, which implied that the gerbils were pre-trained identically for PAT (Figure 4C).

Among all sham groups, latency time in PAT at 5 days after sham operations was not significantly different (Figure 4C). In the TI/vehicle group, a significant shortness in latency time at 5 days after TI was observed compared with that in the sham/vehicle group (Figure 4C). However, in the TI/decursin and TI/AGNE groups, the latency time was significantly lengthened compared with that in the TI/vehicle group (Figure 4C).

### 2.4. Neuroprotection by Decursin and AGNE

**Figure 5:**
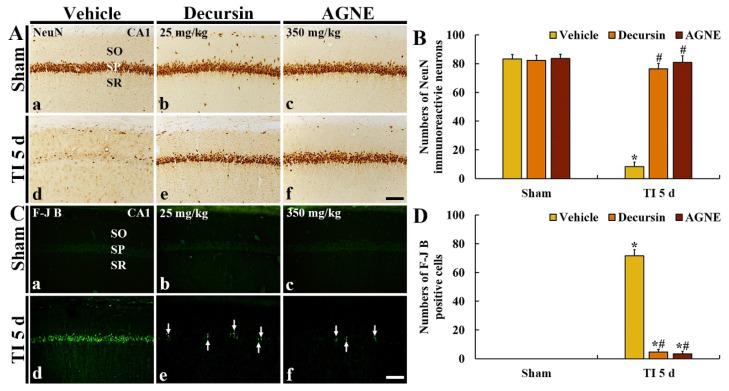
(**A**,**C**) Immunohistochemistry with NeuN (**A**) and histofluorescence with fluoro-Jade B (F-J B) (**C**) in CA1 of the sham/vehicle, sham/decursin, and sham/AGNE groups (**a**–**c**), and TI/vehicle, TI/decursin, and TI/AGNE groups (**d**–**f**) at 5 days after TI. In the TI/vehicle group, NeuN immunoreactive pyramidal neurons are rarely found, whereas many F-J B positive pyramidal neurons are observed in the stratum pyramidale (SP) at 5 days after TI. However, in the TI/decursin and TI/AGNE groups, many NeuN immunoreactive and a few F-J B positive pyramidal neurons (arrows) are observed in the SP. (**B**,**D**) The mean numbers of NeuN immunoreactive (**B**) and F-J B positive (**D**) pyramidal neurons. SO, stratum oriens; SR, stratum radiatum. Scale bar = 100 μm. The bars indicate the means ± SEM (*n* = 7 per group, * *p* < 0.05 versus sham/vehicle group, # *p* < 0.05 versus TI/vehicle group).

To investigate neuroprotective effects of decursin and AGNE in CA1, at 5 days after TI, we carried out immunohistochemistry with neuronal nuclei-specific protein (NeuN, a marker for neuron) and histofluorescence with fluoro-Jade B (F-J B, a marker for neuronal degeneration) (Figure 5).

#### 2.4.1. NeuN Immunoreactive Neurons

In all sham groups, pyramidal neurons (about 83 cells/250 μm^2^) located in CA1 (called CA1 pyramidal neurons) showed strong NeuN immunoreactivity (Figure 5(Aa–Ac)). In the TI/vehicle group, NeuN immunoreactive pyramidal neurons were significantly decreased (about 8 cells/250 μm^2^) at 5 days after TI compared with those found in the sham/vehicle group (Figure 5(Ad,B)).

On the other hand, in the TI/decursin and TI/AGNE groups, most of the CA1 pyramidal neurons survived compared with those in the TI/vehicle group at 5 days after TI, showing that the numbers of the two groups were about 76 and 81 cells/250 μm^2^, respectively (Figure 5(Ae,Af,B)).

#### 2.4.2. F-J B Positive Cells

In all sham groups, F-J B positive cells were not found in CA1 (Figure 5(Ca–Cc)). In the TI/vehicle group, many F-J B positive pyramidal cells (about 72 cells/250 μm^2^) were observed in the stratum pyramidale at 5 days after TI (Figure 5(Cd)).

On the other hand, in the TI/decursin and TI/AGNE groups, F-J B positive pyramidal cells were rarely found (about 4 and 3 cells/250 μm^2^, respectively) at 5 days after TI compared with those in the TI/vehicle group, indicating that most of the CA1 pyramidal neurons were significantly conserved after TI (Figure 5(Ce,Cf,D)).

### 2.5. Attenuation of TI-Induced BBB Leakage (IgG) by Decursin and AGNE

**Figure 6:**
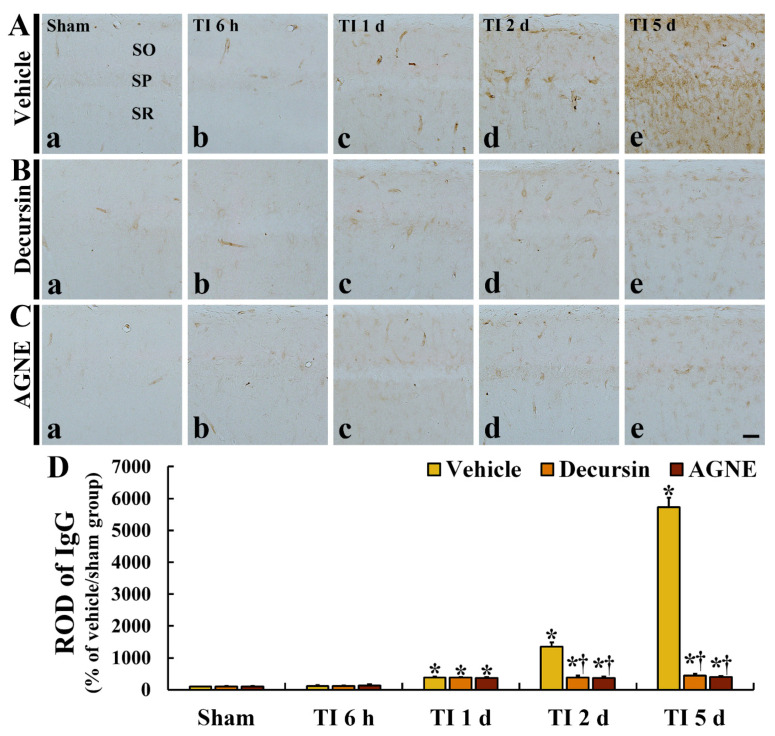
(**A**–**C**) IgG immunoreactivity in CA1 of the vehicle treated (**Aa**–**Ae**), decursin treated (**Ba**–**Be**), and AGNE treated (**Ca**–**Ce**) groups at 6 h (all **b**), 1 day (all **c**), 2 days (all **d**), and 5 days (all **e**) after TI. In the TI/decursin and TI/AGNE groups, IgG immunoreactivity at 2 and 5 days after TI is significantly lower than that in the TI/vehicle group. (**D**) Relative optical density (ROD) of IgG immunoreactivity in CA1. Scale bar = 100 μm. The bars indicate the means ± SEM (*n* = 7 at each time after TI, * *p* < 0.05 vs. sham/vehicle group, # *p* < 0.05 vs. TI/vehicle, and † *p* < 0.05 vs. corresponding TI/vehicle group).

We conducted immunohistochemistry for immunoglobulin G (IgG, an indicator of extravasation) in CA1 to investigate the leakage of the BBB due to TI (Figure 6).

In all sham groups, IgG immunoreactivity was hardly detected in CA1 parenchyma, showing that IgG immunoreactivity was shown inside blood vessels (Figure 6(Aa,Ba,Ca)). In the TI/vehicle group, IgG immunoreactivity in CA1 parenchyma was gradually and markedly increased with time after TI, showing that relative optical density (ROD) of IgG immunoreactivity was 1353% at 2 days and 5723% at 5 days after TI compared with that in sham/vehicle group (Figure 6(Ac–Ae,D)).

In the TI/decursin and TI/AGNE groups, IgG immunoreactivity was not significantly increased in CA1 parenchyma after TI, showing that ROD of IgG was 28.6% and 26.9% at 2 days and 7.8% and 6.9% at 5 days compared with that in the TI/vehicle group (Figure 6(Bd,Be,Cd,Ce,D)).

### 2.6. Attenuation of TI-Induced Astrocyte Endfeet (AEF) Damage by Decursin and AGNE

**Figure 7:**
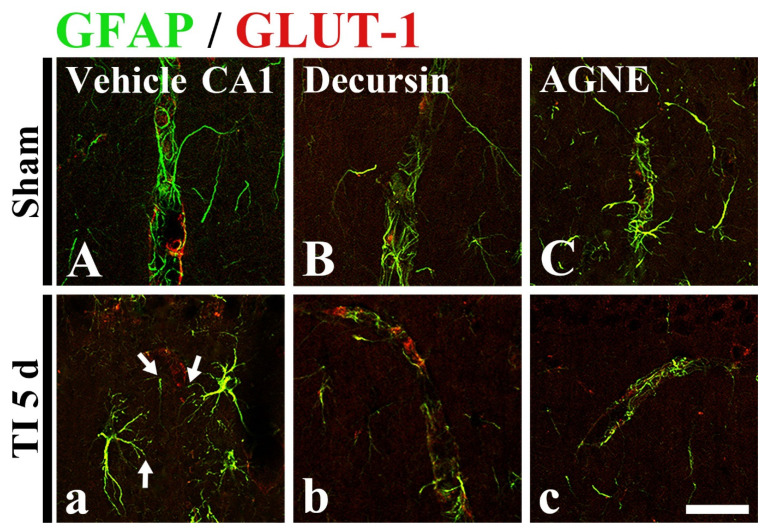
Double immunohistofluorescence for GFAP (green) and GLUT-1 (red) in CA1 of the sham groups (**A**–**C**), TI/vehicle (**a**), TI/decursin (**b**), and TI/AGNE (**c**) at 5 days after TI. In the TL/vehicle group, GFPA immunoreactive endfeet are damaged (arrows). In the TI/decursin and TI/AGNE groups, GFAP and GLUT-1 immunoreactive structures are well arranged like those in the sham groups. Scale bar = 50 μm.

To examine the damage of AEF, which is a component of the BBB, following TI, double immunofluorescence was performed using glial fibrillary acidic protein (GFAP, a marker for astrocyte) and glucose transporter 1 (GLUT-1, a marker for endothelial cells) antibodies in CA1 (Figure 7).

In all sham groups, GFAP immunoreactive astrocytes (green) and GLUT-1 immunoreactive endothelial cells (red) were well distinguished, namely, the endfeet of GFAP immunoreactive astrocytes wrapped GLUT-1 immunoreactive structures (Figure 7A–C). In the TI/vehicle group, GFAP immunoreactive structures (AEF) did not well wrap GLUT-1 immunoreactive structures at 5 days after TI, indicating that the BBB structure was disrupted following TI (Figure 7a).

On the other hand, in the TI/decursin and TI/AGNE/TI groups, the distribution pattern of GFAP immunoreactive AEF and GLUT-1 immunoreactive structures (endothelial cells) was similar to that in the sham groups, indicating that the AEF well wrap the endothelial cells (Figure 7b,c).

## 3. Discussion

It is well accepted that the pattern and degree of neuronal loss/death in brains following ischemia-reperfusion injury are independently reliant on various ischemic qualifications, such as animal models, duration of ischemic period, and body temperature. For example, the different aspects of neuronal damage are shown depending on the duration of ischemic period even in the identical animal models of cerebral ischemia. In detail, it was reported that infarct (necrotic brain tissue) was not shown, and instead the degree of selective neuronal loss/death was differently found in the caudate-putamen of rat models of transient focal brain ischemia (TFBI) induced by middle cerebral artery occlusion (MCAO) for 15 and 30 min [5]. Moreover, rat models of TFBI induced by MCAO for 1 h or more developed infarct, which is commonly visualized by 2, 3, 5-triphenyltetrazolium chloride (TTC) staining assay [24]. In the instance of a gerbil model of transient forebrain ischemia for 5 min, infarct was not developed but selective DND occurred in the hippocampus, which was easily detected by F-J B histofluorescence [4,8,22]. In our current study, DND was well confirmed in pyramidal neurons located in the hippocampal CA1 following 5-munute TI in the forebrain.

As described above, the pattern and degree of neuronal loss/death in brains following ischemia-reperfusion injury are independently reliant on various ischemic qualifications. In this regard, until now, positive control for ischemic stroke has been not established; thus, many studies have applied various neuroprotective agents as a positive control which can treat TI. For example, edaravone (Radicava^®^), which is used for help with recovery following a stroke and to treat amyotrophic lateral sclerosis, was utilized as an agent for a positive control against transient focal cerebral ischemia (TFCI) in rats [25]. In addition, Ai et al. (2017) used nimodipine, which is a calcium channel blocker and used in preventing vasospasm secondary to subarachnoid hemorrhage, for a positive control which treated MCAO-induced TFCI [26]. 

Until now, studies have reported neuroprotective materials against ischemic insults showing that their protective effects display different efficacies depending on the time point of administration (before or after ischemic-reperfusion). For example, pre-treatment with 25 mg/kg lacosamide, a novel antiepileptic drug, exerts strong neuroprotective effects following 5 min TI in gerbils, but post-treatment with the same dose after TI displayed a significant low effectiveness compared with the effects by the pre-treatment [27]. These results imply that the administration of lacosamide can be used for protection (prevention) against ischemic injury instead of therapy after ischemic insults. Additionally, Lee et al. (2011) demonstrated that pre-treatment with 20 mg/kg escitalopram, a selective serotonin re-uptake inhibitor, prevented neuronal death/loss in the hippocampus of a gerbil model of 5 min TI and post-treatment with 30 mg/kg escitalopram saved the hippocampal neurons from TI. Their results indicate that, in the instance of post-treatment, a higher dose of escitalopram needs to accomplish therapeutic effects compared with preventive (protective) effects by pre-treatment [28]. 

Based on the above-mentioned precedent studies, the types of animal models of brain ischemic insults and the administration time of AGNE must affect the results of prevention and therapy in experiments on ischemic insults [8,22,27,28]. Oh et al. (2015) showed that post-treatment with 100 mg/kg AGNE significantly attenuated infarct in a rat model of MCAO-induced TFCI [22]. In our current study, using a gerbil model of 5 min TI, post-treatment with 350 mg/kg AGNE after TI effectively saved the CA1 pyramidal neurons in the hippocampus from TI. Furthermore, we quantified decursin contained in AGNE by HPLC, and the decursin was contained by 7.3 ± 0.2% versus the total amount of the AGNE. Based on this HPLC result, we examined the therapeutic effect of decursin by post-treatment with 25 mg/kg, which is a corresponding dosage of the decursin contained in AGNE, which saved CA1 pyramidal neurons form the TI.

It is well addressed that the hippocampus is importantly connected with learning and memory function [29,30]. Neuronal loss/death in the hippocampus with TI damage brings dysfunction in behavioral outcomes when PAT (short-term and long-term memory function) and 8-ARMT (spatial memory function) are evaluated [31,32,33]. Accumulating evidence has shown that materials possessing neuroprotective potential ameliorate cognitive dysfunction following ischemic insults using behavioral tests [8,34,35]. Our present results showed that the latency time in PAT was lengthened and the number of errors in 8-ARMT was increased when the PAT and 8-ARMT were done. However, the learning and memory dysfunction was ameliorated by post-treatment with 350 mg/kg AGNE and 25 mg/kg decursin. This attenuation might be resulted from the survival of the CA1 pyramidal neurons by post-treatment with AGNE and decursin.

In our current study, the post-treatment with AGNE and decursin maintained BBB integrity showing that AEF enclosing microvessels remained in the ischemic CA1 at 5 days after TI. Additionally, the extravasation of IgG into the CA1 parenchyma was prevented by post-treatment with AGNE and decursin. The structural components of the BBB involve endothelial cells and their linkages (tight junctions), pericytes, AEF, and extracellular membranes [36]. In particular, a highly selective semi-permeability of the BBB is achieved by a close connection between AEF and endothelial cells [37]. Accumulating experimental data show that BBB disruption following ischemic insults leads to vascular leakages due to increased permeability of the BBB. For example, Zhang et al. (2017) demonstrated that BBB permeability was increased following ischemic insults, showing that Evans blue dye invaded into brain parenchyma in a rat model of MCAO-induced TFCI [24]. In addition, some studies showed that leaked serum proteins including IgG and albumin into the perivascular space and brain parenchyma was found following 5 min TI in gerbils [38,39,40]. In particular, Ahn et al. (2018) reported that the proportion of microvessels enclosed by AEF was significantly decreased after 5 min TI in gerbils [33]. 

In conclusion, our current findings revealed that post-treatment with AGNE and decursin saved neurons from ischemic injury in the hippocampus induced by 5 min TI in gerbils and ameliorated TI-induced memory impairment. In addition, the post-treatment with AGNE and decursin after TI inhibited BBB leakage and attenuated AEF damage in the ischemic CA1, which might closely contribute to the survival of the neurons from TI injury. Based on these results, we propose that AGNE and decursin can be utilized as a pharmaceutical composition to develop a therapeutic strategy for brain ischemic injury.

## 4. Materials and Methods

### 4.1. Animals and Protocol Used in This Experiment

Male Mongolian gerbils (*n* = 182; body weight, 85 ± 5 g; 6 months of age) were provided by Experimental Animal Center in Kangwon National University (Chuncheon, Korea). They were housed in standard conditions with 3–5 gerbils per cage with adequate conditions (room temperature, 23 ± 0.5 °C; relative humidity, 55 ± 5%; 12:12 light/dark cycle) with freely accessible pellet feed obtained from DBL Co Ltd. (Chungbuk, Korea) and water.

Experiments in this study were performed in accordance with the guidelines of the “Current International Laws and Policies” from the “Guide for the Care and Use of Laboratory Animals” [41], and the protocol of this experiment was approved by the “Institutional Animal Care and Use Committee (IACUC)” in Kangwon National University (approval no., KW-200113-1, approved on 18 February 2020).

### 4.2. Experimental Groups

They were divided into ten groups: (1) sham/vehicle (*n* = 14), (2) TI/vehicle group (*n* = 28), (3) sham/AGNE (200 mg/kg) (*n* = 14), (4) TI/AGNE (200 mg/kg) group (*n* = 14), (5) sham/AGNE (350 mg/kg) (*n* = 14), (6) TI/AGNE (350 mg/kg) group (*n* = 28), (7) sham/AGNE (400 mg/kg) (*n* = 14), (8) TI/AGNE (400 mg/kg) group (*n* = 14), (9) sham/decursin (25 mg/kg) (*n* = 14), and (10) TI/decursin (25 mg/kg) (*n* = 28).

To determine the dosage of AGNE showing therapeutic effect after TI, the gerbils in all sham groups and in the groups treated with 200, 350, and 400 mg/kg AGNE were sacrificed at 0 and 5 days after TI. In order to examine the mechanisms of the effects, the gerbils in the TI/AGNE (350 mg/kg) and TI/decursin (25 mg/kg) groups were sacrificed at 0, 6 h, 1 day, 2 days, and 5 days after TI.

### 4.3. Decursin Preparation, and AGNE Extraction and Its Qualitative Analysis

Decursin, a coumarin-derived phenolic compound, is regarded as one of the major ingredients of AGNE [42]. The decursin used in this study was purchased from ChemFaces Biochemical Co Ltd. (Cat. No., CFN9859; Wuhan, China). As described previously [20], AGNE was manufactured by KGC Yebon Co Ltd. (Chungju, Chungbuk, Korea) with a standardized extracting process. AGN were farmed in Chuncheon (Gangwon, Korea), and their roots were collected and washed with pure water followed by dehydration. Thereafter, using a grinder (IKA M20, IKA, Staufen, Germany), the dried AGN roots were powderized. Five times the volume of ethanol solution (98% *v/v*) versus the powder was used as an extraction solvent. The powder was extracted for 4 h at 55 ± 5 °C. Next, the extract was filtered using a Whatman No 1 filter (Whatman Ltd., Maidstone, Kent, UK) and concentrated with a vacuum evaporator at 55 ± 5 °C. The final weight of the AGNE was ten percent, compared with the initial weight. Finally, the AGNE was lyophilized and stored at −20 °C.

High-performance liquid chromatography (HPLC) was done to qualitatively analyze the AGNE according to a method by Lee et al. (2020) [20]. In short, decursin (0.01 g for standard sample) and AGNE (1 g for test sample) were respectively dissolved in 50 mL of methanol solution (50% *v/v*; in distilled water). The standard and test samples (10 μL respectively) were chromatographed with 1.0 mL/min of flow rate using Waters 2695 Separation Module HPLC System of Water Co (Milford, MA, USA) and stainless column (inner diameter, 4.6 mm; length, 250 mm; Sunfire™ C_18_) of Water Co (Milford, MA, USA) filled with octadecylsilyl silica gel (diameter, 5 μm). A (acetonitrile) and B (distilled water) solutions were used for mobile phases with a concentration gradient as follows: 0 min (20% A), 3 min (20% A), 8 min (30% A), 18 min (30% A), 19 min (50% A), 40 min (50% A), 41 min (90% A), and 50 min (90% A). Decursin contained in AGNE was detected using a Waters 996 Photodiode Array Detector with 330 nm of wavelength.

### 4.4. Induction of TI

The gerbils of the sham and TI groups were given sham and TI operations according to our published method [4]. Briefly, the gerbils were anesthetized by mixture of isoflurane (induction, 2.5%; maintain, 2.0%) of Hana Pharmaceutical Co Ltd. (Seoul, Korea) in 33% oxygen and 67% nitrous oxide using an inhaler [43]. The ventral skin of the neck was shaved, and midline incision was made. Thereafter, left and right common carotid arteries (CCA) were ligated with non-traumatic aneurysm clips (0.69 N; Yasargil FE 723K) (Aesculap, Tuttlingen, Germany) for 5 min. The stopping of blood circulation was observed in the central artery of the retina, which is a branch of the internal carotid artery that supplies blood to the forebrain, using an ophthalmoscope (HEINE K180^®^, Heine Optotechnik) (Herrsching, Germany). After CCA ligation for 5 min, the clips were removed, and the incised skin was sutured with 3-0 suture silk (Ethicon Inc, Somerville, NJ, USA). During the TI surgical procedure, body temperature was monitored in real-time with a rectal temperature probe (TR-100) of Fine Science Tools (Foster City, CA, USA) and maintained at normothermic condition (37 ± 0.5 °C). The gerbils of the sham groups were subjected to the identical surgical procedure without CCA ligation.

### 4.5. Post-Treatment with Decursin and AGNE

The gerbils of the decursin and AGNE treated groups were orally administrated decursin and AGNE at 30 min after sham and TI operations. In detail, decursin and AGNE were dissolved in saline (0.85% NaCl *w*/*v*). To obtain the dosage of AGNE showing neuroprotection, 200, 350, and 400 mg/kg were used with reference to a paper by Oh e al. (2015) [22]. Since post-treatment with 350 mg/kg AGNE showed a neuroprotective effect against TI in the present study, the dosage of decursin was chosen as 25 mg/kg, which is a corresponding dosage of the decursin contained in AGNE (about 7.3 ± 0.2%). The gerbils ingested decursin and AGNE immediately after TI operations using a curved feeding needle (length, 5.08 cm; 18 gauge) (LafeberVet, Illinois, IL, USA).

### 4.6. Tests for Cognitive Function

#### 4.6.1. 8-ARMT

The 8-ARMT was performed in order to examine change in spatial memory following TI according to previously published protocols [8,33] with some modifications. An apparatus for the maze was utilized, which consisted of an opaque acryl board (central platform; diameter, 20 cm) with 8 radially extended arms (width, 5 cm; height, 9 cm; length, 35 cm) of Stoelting Co. (Illinois, IL, USA). The gerbils were pre-trained once a day for three days before TI. The substantive test was carried out daily for 5 days from 1 day after TI. In detail, we put a feed pellet of DBL Co Ltd. (Chungbuk, Korea) at the end of each arm, and each gerbil was placed onto the central platform. The numbers of the errors were counted every time the gerbil went into an arm that the gerbil already entered before. Each test was finished when the gerbil consumed all feed pellets.

#### 4.6.2. PAT

Short term memory was evaluated through PAT according to previously described methods [7,32] with minor modifications. In short, the Gemini Avoidance System (GEM 392) of San Diego Instruments (San Diego, CA, USA) was used. This system consists of two compartments which communicate through a vertically sliding door in the middle. The experimental session was divided into two phases (training and test session) at one day before TI and five days after TI. Twenty minutes after the training session, the substantive trial was performed. Namely, in the training session, the gerbil was freely allowed to explore light and dark rooms for one minute while the sliding door was opened. Thereafter, when the gerbil entered the dark room, the door was closed, and the gerbil was given an electric foot-shock (0.5 mA for 5 s) from a steel grid located on the floor. In the test session, the gerbil was placed in the light room, and the latency time (seconds) to enter the dark room was recorded within three minutes. 

### 4.7. Preparation of Histological Sections

For histological or histopathological examination, brain tissues containing the hippocampus were prepared as described previously [44,45]. The gerbils were deeply anesthetized by intraperitoneal injection of pentobarbital sodium (90 mg/kg) (JW Pharmaceutical Co Ltd., Seoul, Korea) [43]. Under the anesthesia, their brains were rinsed by transcardial perfusion with saline and fixed with 4% paraformaldehyde solution (in phosphate buffer, PB; pH 7.4). Thereafter, the brains were removed and stored in the same fixative for 4 h at room temperature. For the section, the fixed brains were infiltrated in 30% sucrose solution (in PB, pH 7.4) for 24 h at room temperature to protect the brains from cryosection. Thereafter, these brains were cut coronally sectioned into 30 μm thickness on sliding microtome (SM2020 R) of Leica (Nussloch, Germany) equipped with a BFS-40MP freezing stage of Physitemp Instruments Inc. (New Jersey, NJ, USA). Representative sections were obtained at the level of −1.8 to 2.7 mm antero-posterior to the bregma by reference to according to the “Brain Atlas of the Mongolian Gerbil (*Meriones unguiculatus*)” [46].

### 4.8. CV Histochemistry

CV histochemical staining was performed according to a published method [47]. Briefly, the sections were mounted onto the gelatin-coated microscopy slides. The sections were stained with 0.1% CV acetate solution (Sigma-Aldrich Co, St. Louis, MO, USA) for 15 min at room temperature and washed in distilled water. After decolorization in 70% ethyl alcohol for a few seconds, the stained sections were dehydrated in 80%, 90%, 95%, and 100% ethyl alcohol, and cleared in xylene. Finally, the slides were mounted with Canada balsam (Kanto Chemical Co Inc, Tokyo, Japan).

To compare CV-stained cells in the hippocampus in all groups, the images of the CV-stained hippocampus were captured using a microscope (BX53) (Olympus, Tokyo, Japan), equipped with a digital camera (DP72) (Olympus, Tokyo, Japan).

### 4.9. F-J B Histofluorescence

Histofluorescence with F-J B was performed to investigate damage/death (loss) of cells in the hippocampus following TI. We used the methods published by Anderson et al. (2005) and Schmued and Hopkins (2000) [48,49] with slight modification. In brief, the sections were mounted onto the microscopy slides coated with gelatin. The sections were incubated in 0.06% potassium permanganate (KMnO_4_) solution (Sigma-Aldrich Co, St. Louis, MO, USA) for 10 min on a rotating stage and rinsed in distilled water for 2 min. Thereafter, the sections were incubated in 0.0004% F-J B solution (Histochem, Jefferson, AR, USA) for 20 min and washed with distilled water. For the reaction of F-J B, the sections were placed on a slide warmer till the sections were fully dried. Finally, the slides were cleared by immersion in xylene (Junsei Chemical Co Ltd., Tokyo, Japan) and coverslipped with dibutyl phthalate polystyrene xylene (DPX) (Sigma-Aldrich Co, St. Louis, MO, USA).

For the count of F-J B positive cells (neurons), five sections per gerbil were selected and analyzed according to a method by Sharma et al. (2007) [44] with some modification. In short, the images of F-J B positive cells were taken using an epifluorescence microscope (BX53) of Olympus (Tokyo, Japan) equipped with a 450–490 nm blue excitation light, and the cells were captured using image capture software (cellSens Standard) (Olympus, Tokyo, Japan). F-J B positive cells were totally counted in 250 μm^2^ at the middle in CA1, and the mean number was calculated using NIH Image 1.59 software (NIH, Bethesda, Maryland, MD, USA).

### 4.10. Immunohistochemistry

For the immunohistochemical studies, the avidin-biotin complex (ABC) method was used according to previously published methods [47,50] with some modification. The prepared sections were rinsed in 100 mM phosphate buffered saline (PBS; pH 7.4). Endogenous peroxidase activity in the sections was blocked with 0.3% hydrogen peroxide (H_2_O_2_) for 20 min at room temperature, and non-specific proteins were blocked through immersing the sections in 5% horse or goat normal serum for 30 min at room temperature. Thereafter, the sections were immunoreacted with each primary antibody—mouse anti-NeuN (1:1000) (Chemicon, Temecula, CA, USA) and rabbit anti-gerbil IgG (1:1000) (Bioss antibodies, Atlanta, GA, USA) for 10 h at 4 °C. Next, the sections were incubated with corresponding biotinylated secondary antibodies—horse anti-mouse IgG (1:250) (Vector Laboratories, Burlingame, CA, USA) and goat anti-rabbit IgG (1:250) (Vector Laboratories) for 90 min at room temperature followed by ABC (1:300) (Vector Laboratories) for 60 min at room temperature. Thereafter, the sections were visualized by 0.06% 3,3′-diaminobenzidine tetrahydrochloride (DAB) solution (Sigma-Aldrich Co, St Louis, MO, USA) in 100 mM PBS containing 0.1% H_2_O_2_. Immediately, the sections were rinsed, dehydrated in 70%–100% ethyl alcohol, and cleared in xylene. Finally, the sections were coverslipped with Canada balsam (Kanto Chemical Co Inc, Tokyo, Japan).

As a negative control, the same tissues were incubated with pre-immune serum except for each primary antibody. In the sections, no immunostained structures were found (data not shown).

To count the number of NeuN immunoreactive neurons, five sections/gerbil were chosen and analyzed as described above (in Section 4.9. F-J B histofluorescence).

To quantify the optical density of IgG immunoreactive structure, five sections/gerbil were taken and observed using a microscope (BX53) equipped with a digital camera (DP72) and image capture software of cellSens Standard (Olympus, Tokyo, Japan). As previously described [51], the captured IgG image was converted into 8-bit grey scale images with a range of 0–255 (from black to white). The image was assessed for grey scale intensity, and the immunoreactive intensity of the average staining was calculated using Image J software (version 1.46) (National Institutes of Health, Bethesda, Maryland, MD, USA). The immunoreactive intensity was relatively presented as a percentage (100% in the sham group).

### 4.11. Double Immunohistofluorescence

Double immunohistofluorescence was performed to distinguish AEF from endothelial cells in the BBB. Primary antibodies used in this experiment were mouse anti-GFAP (a marker for astrocytes) (1:1000) (Chemicon, Temecula, CA, USA) and rabbit anti-GLUT-1 (a marker for endothelial cells) (Chemicon). As described previously [38], the sections were immunoreacted with a mixture of Alexa Fluor^®^ 488-conjugated donkey anti-mouse IgG (1:500) (Invitrogen, Waltham, MA, USA) and Alexa Fluor^®^ 546-conjugated goat anti-rabbit IgG (1:500) (Invitrogen, Waltham, MA, USA). The immunoreacted sections were mounted onto slide glasses and dehydrated in a dry oven of WiseVen^®^ WOC High Clean Air Oven (Daihan Scientific Co Ltd., Gangwon, Korea). Finally, the sections were coverslipped with Canada balsam (Kanto Chemical Co Inc, Tokyo, Japan).

The double immunoreaction (GFAP/GLUT-1) was observed using confocal MS (LSM510 META NLO) from Carl Zeiss (Oberkochen, Germany) located in the Korea Basic Science Institute Chuncheon Center (Chuncheon, Kangwon, Korea).

### 4.12. Statistical Analysis

Data presented in this study were displayed as the means ± standard error of mean (SEM). All data were statistically analyzed using GraphPad Prism software (version 5.0) of GraphPad Software (La Jolla, CA, USA). The significant differences of the mean among the experimental groups were analyzed by two-way analysis of variance (ANOVA) with a post hoc Tukey’s test for all pairwise multiple comparisons. A lower than 0.05 of *p* value designated the significant differences.

## Figures and Tables

**Table 1:** Ingredients of *Angelica gigas* Nakai root extract (AGNE).

Type		Ingredients
Coumarin derivatives	Major ingredients	Decursin
		Decursinol angelate
		Decursinol
	Other ingredients	(2′′R,3′′R)-epoxyangeloyldecursinol, (2′′S,3′′S)-epoxyangeloyldecursinol, 4′′-Hydroxytigloyldecursinol, 4′′-hydroxydecursin, columbianetin-O-β-D-glucopyranoside, Marmesinin, Nodakenin, etc.
Saccharides	Polysaccharide	Angelan (peptic polysaccharide)
	Monosaccharides	Arabinose, Galactose, Galacturonic acid (sugar acid), etc.
Polyacetylene		18-acetoxy-octadeca-1,9-dien-4,6-diyn-3,8-diol, Octadeca-1,9-dien-4,6-diyn-3,8,18-triol, etc.

## Data Availability

The data presented in this study are available on request from the corresponding author.

## Sample Availability

Samples of the compounds are not available from the authors.

## Abbreviations

8-ARMT	8-arm radial maze test
AEF	astrocyte endfeet
AGNE	*Angelica gigas* Nakai root extract
BBB	blood–brain barrier
CA	cornu ammonis
CCA	common carotid artery
CNS	central nervous system
CV	cresyl violet
DND	delayed neuronal death
F-J B	fluoro-Jade B
GFAP	glial fibrillary acidic protein
GLUT-1	glucose transporter 1
HPLC	high-performance liquid chromatography
IgG	immunoglobulin G
NeuN	neuronal nuclei-specific protein
PAT	passive avoidance test
ROS	reactive oxygen species
TI	transient ischemia
